# Medication-Associated Xerostomia and Oral Symptom Burden in Midlife Women: A Drug Safety Perspective Across the Menopausal Transition

**DOI:** 10.3390/ph19101577

**Published:** 2026-10-05

**Authors:** Ana-Cristiane Dragomir, Radu Dumitru Moleriu, Ruxandra-Cristina Marin, Alina Ramona Buzatu, Călin Muntean, Teodora Piroș, Iulia Najette Crintea, Maria Sala-Cirtog, Edward Seclaman, Marilena Dinuti

**Affiliations:** 1 Doctoral School, “Victor Babeș” University of Medicine and Pharmacy, 300041 Timisoara, Romania; cristiane.dragomir@umft.ro; 2 Discipline of Biochemistry, Department of Biochemistry and Pharmacology, Faculty of Medicine, Victor Babeș University of Medicine and Pharmacy, 2 Eftimie Murgu Square, 300041 Timisoara, Romania; mariasala.cirtog@umft.ro (M.S.-C.); eseclaman@umft.ro (E.S.); dinuti.marilena@umft.ro (M.D.); 3 Department III, Functional Science, Discipline of Medical Informatics and Biostatistics, “Victor Babeș” University of Medicine and Pharmacy, 300041 Timisoara, Romania; radu.moleriu@umft.ro (R.D.M.); cmuntean@umft.ro (C.M.); teodora.piros@student.umft.ro (T.P.); 4 Discipline of Pharmacology, Clinical Pharmacology and Pharmacotherapy, “Carol Davila” University of Medicine and Pharmacy, 050474 Bucharest, Romania; 5 Doctoral School of Biological and Biomedical Sciences, University of Oradea, 410087 Oradea, Romania; 6 Department of Surgery I, Faculty of Medicine, Victor Babeș University of Medicine and Pharmacy, 2 Eftimie Murgu Square, 300041 Timisoara, Romania; iulia.crintea@umft.ro; 7 Emergency Department, Emergency Clinical Municipal Hospital Timisoara, 300079 Timisoara, Romania

**Keywords:** xerostomia, dry mouth, adverse drug reaction, drug safety, pharmacovigilance, menopause, midlife women, medication burden, propensity score, two-part model

## Abstract

**Background/Objectives:** Medication-associated xerostomia is a clinically relevant adverse effect, but its contribution to oral symptom burden in midlife women remains insufficiently characterized. This study investigated the association between xerogenic medication exposure and xerostomia burden across the menopausal transition and whether this association extended to broader oral symptoms. **Methods:** This cross-sectional study included 306 Romanian women aged 45–65 years. Medication exposure was assessed in two complementary ways: a medication-list-derived definition, in which reported medicines were coded into therapeutic classes with recognized xerogenic potential (n = 54; 17.6%), regarded as the pharmacological reference measure, and participant-reported use of medication potentially associated with dry mouth (n = 39; 12.7%), which additionally reflects patient recognition of xerogenic exposure. Outcomes were a six-item xerostomia symptom score (XSS-6; 0–18) and a nine-item oral symptom score (OSS-9; 0–27), both derived from the MMBQ-44. Associations were evaluated using two-part models adjusted for age, menopausal stage, body mass index, smoking, medication burden, and psychological symptom burden, with complementary sensitivity analyses. **Results:** Xerostomia symptoms were more frequent among women reporting xerogenic medication (94.9% vs. 71.5%; unadjusted OR 7.36, 95% CI 1.73–31.30), and participant-reported exposure remained associated with greater XSS-6 severity after full adjustment (+1.28 points, 95% CI 0.22–2.34). With the medication-list-derived definition, unadjusted associations were present (rate ratio 1.50, 95% CI 1.08–2.10) but attenuated to near-null estimates after full adjustment (severity difference 0.06 points; rate ratio 1.17, 95% CI 0.79–1.71). Psychological symptom burden was independently associated with both outcomes, and no independent association with OSS-9 was observed for either exposure definition. Agreement between definitions was moderate (85.3%; Cohen kappa 0.43); only 24/54 (44.4%) women identified from medication lists recognized taking medication capable of causing dry mouth. **Conclusions:** Pharmacologically defined xerogenic exposure showed weak independent associations with xerostomia burden after full adjustment, whereas the stronger associations observed for participant-recognized exposure may partly reflect symptom-driven awareness and should be read as an upper bound. Limited recognition of xerogenic exposure points to a medication-safety communication gap. Prospective studies with verified medication exposure, dose and duration data, and objective salivary measurements are needed to clarify causality.

## 1. Introduction

Xerostomia (the subjective sensation of dry mouth) is a well-recognized medication-associated adverse effect: several hundred medicines across more than fifty therapeutic classes have documented xerostomising potential, including antidepressants, anxiolytics, antihistamines, diuretics, and several antihypertensive classes [1,2,3]. Beyond discomfort, chronic oral dryness may compromise multiple protective and functional roles of saliva and is associated with dental caries, mucosal lesions, dysphagia, taste disturbance, and impaired speech, with measurable consequences for nutrition and quality of life [4,5]. But xerostomia is a patient-reported symptom and is not synonymous with objectively measured hyposalivation; the two may coexist but do not necessarily identify the same individuals.

Population studies consistently show that perceived dry mouth increases with age and, at every age, with the number of medicines taken. In a large Swedish population sample, dry-mouth prevalence rose steeply with pharmacotherapy in both sexes and was higher in women [6]; longitudinal data in older adults confirmed that starting xerostomising medication precedes the onset of dry mouth [7], and a meta-analysis quantified markedly increased odds of dry mouth for users of several common drug classes [8]. Medication burden is therefore particularly relevant to xerostomia because salivary effects may accumulate across therapies with different pharmacological mechanisms, making attribution to a single agent difficult in patients receiving multiple medications. From a drug-safety perspective, this creates a clinically important distinction between identifying an individual xerogenic medicine and evaluating the patient’s overall xerogenic medication burden.

Midlife women constitute a particularly relevant population in which to examine this relationship because medication exposure coincides with physiological and symptomatic changes across the menopausal transition. Menopause has been associated with oral dryness, burning symptoms, and mucosal changes [9,10,11,12]; oestrogen receptors are expressed in salivary glands and oral epithelium [13], and salivary oestrogen levels relate to the intensity of oral dryness in menopausal women [14]. However, oral symptoms during this period are multifactorial and cannot be attributed to hormonal changes alone. Age, systemic conditions, medication exposure, psychological symptoms, and oral-health status may contribute simultaneously, complicating attribution of dry-mouth complaints to either menopause or pharmacotherapy [15].

This overlap is increasingly relevant as chronic pharmacotherapy becomes established during midlife. Cardiovascular, metabolic, endocrine, and psychological conditions may require long-term treatment with antihypertensive, lipid-lowering, thyroid, antidiabetic, or psychotropic medications, several of which have direct or indirect potential to influence salivary function. Consequently, medication-associated oral dryness should not be regarded exclusively as a geriatric problem: potentially xerogenic exposure may already be present during the menopausal transition, when competing non-pharmacological determinants of oral symptoms are also prominent [16].

Subjective oral dryness can be measured reliably with the Xerostomia Inventory (XI) and its shortened versions [17,18], while the broader consequences of oral conditions for daily life are captured by the Oral Health Impact Profile (OHIP) family of instruments [19]. Maintaining this distinction is important when investigating medication safety because a specific association with xerostomia does not necessarily imply a comparable association with broader oral-health burden. Subjective dry mouth is also strongly linked to anxiety, depression, and stress [20], so any assessment of medication effects on oral symptoms must account for psychological burden as a potential confounder rather than attributing perceived dryness solely to pharmacological exposure.

Despite the established xerogenic potential of many commonly used medicines, the independent contribution of medication exposure to xerostomia specifically in midlife women remains insufficiently characterized. Existing evidence has largely focused on older or highly medicated populations, individual drug classes, or oral manifestations of menopause, leaving uncertainty about whether medication exposure contributes to a specific dry-mouth burden after menopausal stage, overall medication burden, and psychological symptoms are considered simultaneously [21]. It is also clinically relevant to distinguish pharmacologically identifiable exposure from patients’ own recognition that their medication may cause dry mouth, because unrecognized adverse effects may be less likely to prompt medication review, counselling, or preventive oral care [22].

The present study is a secondary analysis of a Romanian cross-sectional cohort recruited for the development and validation of the 44-item Multidimensional Menopausal Burden Questionnaire (MMBQ-44) [23]. From a pharmacovigilance perspective, characterising adverse-effect burden in special populations under real-world conditions complements spontaneous-reporting systems [24]. Contemporary pharmacovigilance increasingly recognizes the complementary value of real-world evidence for contextualizing suspected or established safety concerns, particularly by providing information on exposed populations, comorbidities, concomitant treatments, and potential confounding factors that cannot be adequately characterized from spontaneous reports alone [25].

Accordingly, this study examined the association between xerogenic medication exposure and xerostomia symptom burden in midlife women after accounting for demographic, menopausal, medication-related, and psychological factors, using two complementary exposure definitions: a medication-list-derived classification based on recognized xerogenic therapeutic classes, treated as the pharmacological reference definition, and participant-reported use of medication potentially associated with dry mouth, treated additionally as a measure of patient recognition of xerogenic exposure. Secondary objectives were to determine whether associations extended to broader oral symptoms and to assess concordance between the two definitions. We hypothesized that associations would be more consistent for the specific xerostomia outcome than for broader oral symptom burden. Given the cross-sectional design, all associations were interpreted as non-causal.

## 2. Results

### 2.1. Participant Characteristics and Medication Exposure

The final analytical sample comprised 306 women, of whom 39 (12.7%) reported taking medication that could cause dry mouth. Compared with women who did not report such exposure, these participants were older (56.8 ± 5.0 vs. 52.9 ± 5.4 years; *p* < 0.001), had a higher BMI (31.4 ± 6.8 vs. 26.8 ± 4.8 kg/m^2^; *p* < 0.001), and were more frequently in the later postmenopausal group (84.6% vs. 62.9%; overall *p* = 0.028). They were also more likely to report no regular physical activity (35.9% vs. 17.6%; *p* = 0.014), higher medication burden (≥3 daily tablets: 56.8% vs. 13.6%; *p* < 0.001), and greater psychological symptom burden (PSY-9: 14.8 ± 9.6 vs. 9.9 ± 8.3; *p* = 0.002). No significant between-group differences were observed for educational attainment, current smoking, alcohol consumption, current menopausal hormone therapy use, or vasomotor symptom burden (Table 1).

Overall, 131 women (43.4% of those with available medication information) reported regular use of at least one medication for a chronic condition. Cardiovascular medications predominated among the therapeutic classes identified from the free-text medication listings. Renin–angiotensin-system agents and β-blockers were the most frequently reported classes, each used by 47 women (15.4% of the overall sample), followed by levothyroxine (n = 28; 9.2%), lipid-lowering agents (n = 15; 4.9%), and antiplatelet agents (n = 13; 4.2%) (Table 2). Because individual participants could report medications from more than one therapeutic class, class frequencies were not mutually exclusive.

Independent coding of the free-text medication listings identified 54 women (17.6%) who reported at least one medication belonging to a class with recognized xerogenic potential [1,8]. This medication-list-derived classification identified a larger group than the participant-reported xerogenic medication measure (17.6% vs. 12.7%). Agreement between the two exposure definitions was 85.3%, with moderate agreement beyond chance (Cohen’s κ = 0.43). The medication-list-derived definition was regarded as the pharmacological reference exposure and was evaluated with the same nested modelling strategy as the participant-reported measure; the participant-reported measure was additionally interpreted as an indicator of patient recognition of xerogenic exposure.

### 2.2. Xerostomia and Oral Symptom Burden According to Participant-Reported Xerogenic Medication Exposure

**Figure 1:**
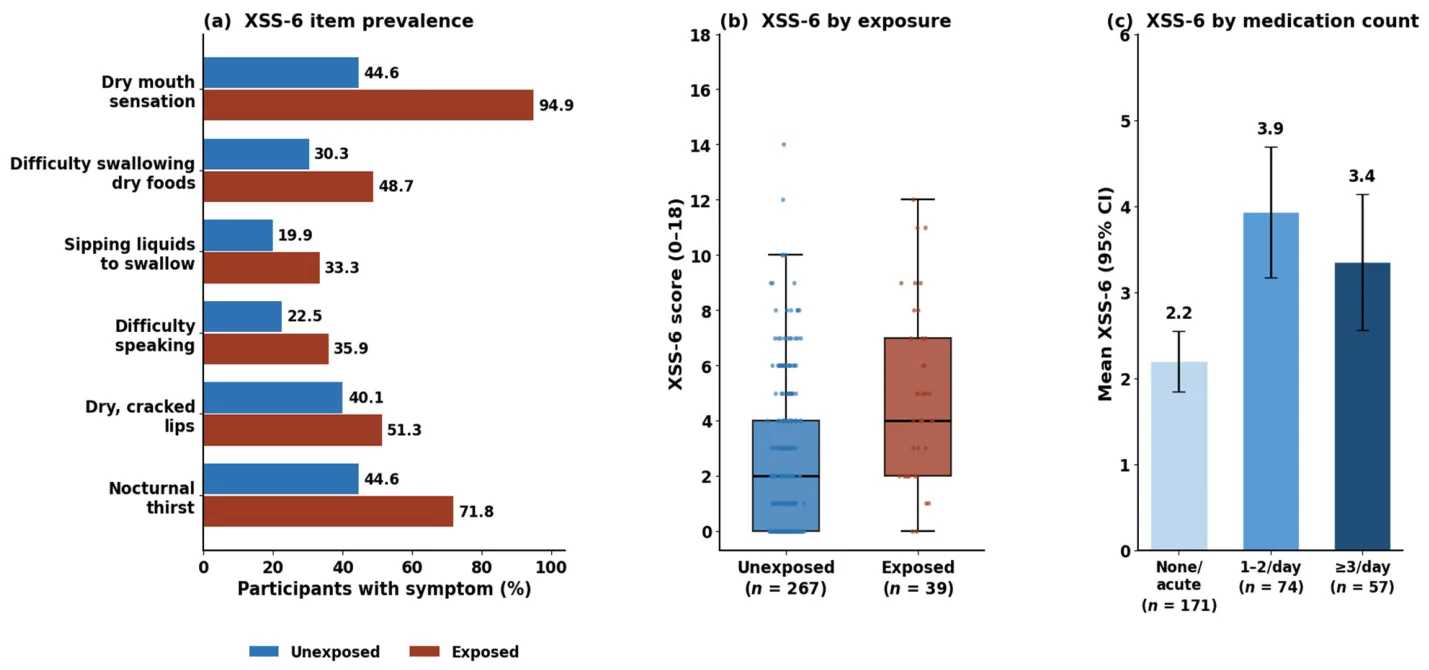
Xerostomia symptom burden according to participant-reported xerogenic medication exposure and overall medication burden. (**a**) Prevalence of individual XSS-6 symptoms according to participant-reported xerogenic medication exposure; (**b**) distribution of XSS-6 scores according to participant-reported xerogenic medication exposure (boxes indicate medians and interquartile ranges; whiskers extend to 1.5 × IQR; points represent individual participants); (**c**) mean XSS-6 scores with 95% confidence intervals according to medication-burden category.

Xerostomia symptoms were more frequent among women reporting use of medication potentially associated with dry mouth. At least one XSS-6 symptom was reported by 37 of 39 exposed women (94.9%), compared with 191 of 267 women (71.5%) who did not report such exposure (*p* = 0.001). Individual xerostomia-related symptoms were also more frequent in the exposed group, with the largest differences observed for subjective dry-mouth sensation (94.9% vs. 44.6%) and nocturnal thirst (71.8% vs. 44.6%) (Table 3; Figure 1a).

Overall XSS-6 scores were higher among women reporting xerogenic medication exposure (mean ± SD, 4.8 ± 3.1; median, 4 [IQR 2–7]) than among those without participant-reported exposure (2.6 ± 2.7; median, 2 [IQR 0–4]; *p* < 0.001) (Table 3; Figure 1b). Higher xerostomia burden (XSS-6 ≥ 6) was observed in 13 exposed women (33.3%) and 45 unexposed women (16.9%; *p* = 0.026).

XSS-6 scores also differed according to medication burden. Mean scores were 2.2 among women reporting no regular chronic medication or acute medication use only, 3.9 among those taking 1–2 daily tablets, and 3.4 among those taking ≥3 daily tablets (Kruskal–Wallis *p* < 0.001). The correlation between medication-burden category and XSS-6 score was positive but weak (Spearman ρ = 0.22) (Figure 1c).

Differences in broader oral-health outcomes were smaller. Women reporting xerogenic medication exposure had higher OSS-9 scores than unexposed women (3.6 ± 3.7 vs. 2.6 ± 3.4; *p* = 0.024), whereas the proportion reporting at least one OSS-9 finding was 76.9% and 60.7%, respectively (*p* = 0.053). The chemosensory symptom score was also higher in the exposed group (1.3 ± 1.7 vs. 0.7 ± 1.2; *p* = 0.048) (Table 3). XSS-6 and OSS-9 scores were moderately correlated (Spearman ρ = 0.54).

### 2.3. Adjusted Association Between Participant-Reported Xerogenic Medication Exposure and Xerostomia Burden

**Figure 2:**
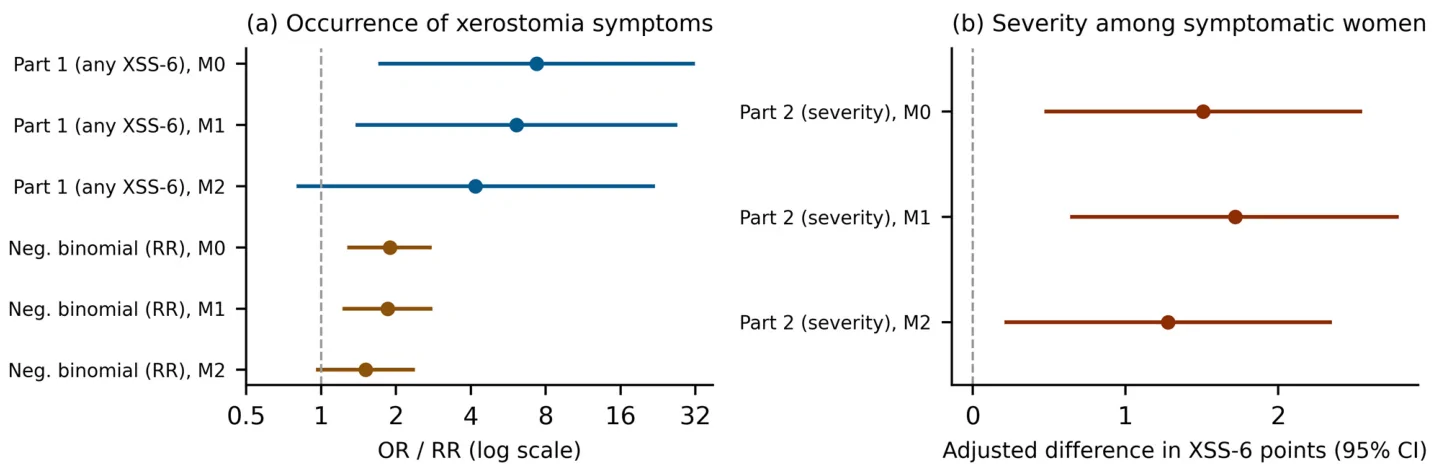
Association between participant-reported xerogenic medication exposure and XSS-6 xerostomia burden across nested adjustment models. The two panels show different types of estimates. (**a**) Relative (multiplicative) estimates: odds ratios (OR) for the presence of any XSS-6 symptom (logistic regression) and rate ratios (RR) for the complete XSS-6 score (negative binomial regression), plotted on a logarithmic x-axis; the dashed line marks the null value of 1. (**b**) Absolute estimates: differences in XSS-6 score among symptomatic women (linear regression), expressed in points on the 0–18 XSS-6 scale (for example, a value of +1 means a score one point higher) and plotted on a linear x-axis; the dashed line marks the null value of 0. M0, unadjusted; M1, adjusted for age, menopausal status, BMI, and current smoking; M2, additionally adjusted for higher medication burden (≥3 daily tablets) and PSY-9 psychological symptom burden. Error bars indicate 95% confidence intervals.

In the unadjusted two-part model, participant-reported xerogenic medication exposure was associated with higher odds of reporting at least one XSS-6 symptom (OR 7.36, 95% CI 1.73–31.30; *p* = 0.007) and with greater xerostomia severity among symptomatic women (β = 1.51 XSS-6 points, 95% CI 0.48–2.54; *p* = 0.004) (Table 4; Figure 2). Adjustment for age, menopausal status, BMI, and current smoking produced similar estimates (M1: OR 6.11, 95% CI 1.40–26.66; *p* = 0.016; β = 1.72, 95% CI 0.65–2.78; *p* = 0.002).

After additional adjustment for higher medication burden and psychological symptom burden (M2), participant-reported xerogenic medication exposure remained associated with greater XSS-6 severity among symptomatic women (β = 1.28 points, 95% CI 0.22–2.34; *p* = 0.018). In contrast, the association with the occurrence of any XSS-6 symptom was attenuated and imprecisely estimated (OR 4.18, 95% CI 0.81–21.62; *p* = 0.088). Only two women in the participant-reported exposure group had an XSS-6 score of zero, resulting in sparse outcome data for the occurrence component of the model.

The complementary negative binomial analysis of the complete XSS-6 score showed a similar pattern. Participant-reported xerogenic medication exposure was associated with a higher XSS-6 score in the unadjusted model (RR 1.89, 95% CI 1.30–2.74; *p* < 0.001) and after adjustment for age, menopausal status, BMI, and current smoking (aRR 1.85, 95% CI 1.24–2.76; *p* = 0.003). Following additional adjustment for higher medication burden and PSY-9, the estimated association was attenuated, with the confidence interval including the null (aRR 1.51, 95% CI 0.97–2.34; *p* = 0.066) (Table 4; Figure 2).

In the fully adjusted two-part model, psychological symptom burden was independently associated with both components of the XSS-6 outcome. Each one-point increase in PSY-9 was associated with higher odds of reporting any xerostomia symptom (OR 1.18, 95% CI 1.12–1.25; *p* < 0.001) and with a 0.15-point higher XSS-6 score among symptomatic women (95% CI 0.11–0.19; *p* < 0.001). Age, menopausal status, BMI, current smoking, and higher medication burden were not independently associated with XSS-6 severity in the fully adjusted model (Table 5).

### 2.4. Association Between Participant-Reported Xerogenic Medication Exposure and Broader Oral Symptom Burden

The association between participant-reported xerogenic medication exposure and the broader OSS-9 outcome was weaker than that observed for XSS-6 (Table 6). In the unadjusted two-part model, participant-reported exposure was associated with higher estimated odds of reporting at least one OSS-9 finding, although the confidence interval included the null (OR 2.16, 95% CI 0.99–4.73; *p* = 0.054). No association with OSS-9 severity among women reporting oral-health findings was observed (β = 0.47 points, 95% CI −0.92 to 1.87; *p* = 0.506). Similarly, the unadjusted negative binomial estimate for the complete OSS-9 score was imprecise (RR 1.41, 95% CI 0.96–2.06; *p* = 0.078).

Estimates were further attenuated after adjustment for age, menopausal status, BMI, and current smoking (M1) and after additional adjustment for higher medication burden and psychological symptom burden (M2). In the fully adjusted model, participant-reported xerogenic medication exposure was not independently associated with the occurrence of any OSS-9 finding (OR 1.14, 95% CI 0.42–3.10; *p* = 0.804) or with OSS-9 severity among women with findings (β = −0.42 points, 95% CI −2.08 to 1.25; *p* = 0.623). The companion negative binomial model yielded a consistent result for the complete OSS-9 score (aRR 1.05, 95% CI 0.67–1.64; *p* = 0.831).

In the fully adjusted occurrence model, later postmenopausal status (OR 2.52, 95% CI 1.16–5.48) and greater psychological symptom burden (OR 1.13 per PSY-9 point, 95% CI 1.09–1.18) were independently associated with the presence of at least one OSS-9 finding. Overall, the unadjusted association between participant-reported xerogenic medication exposure and broader oral symptom burden was not supported after multivariable adjustment.

### 2.5. Pharmacological and Sensitivity Analyses

**Figure 3:**
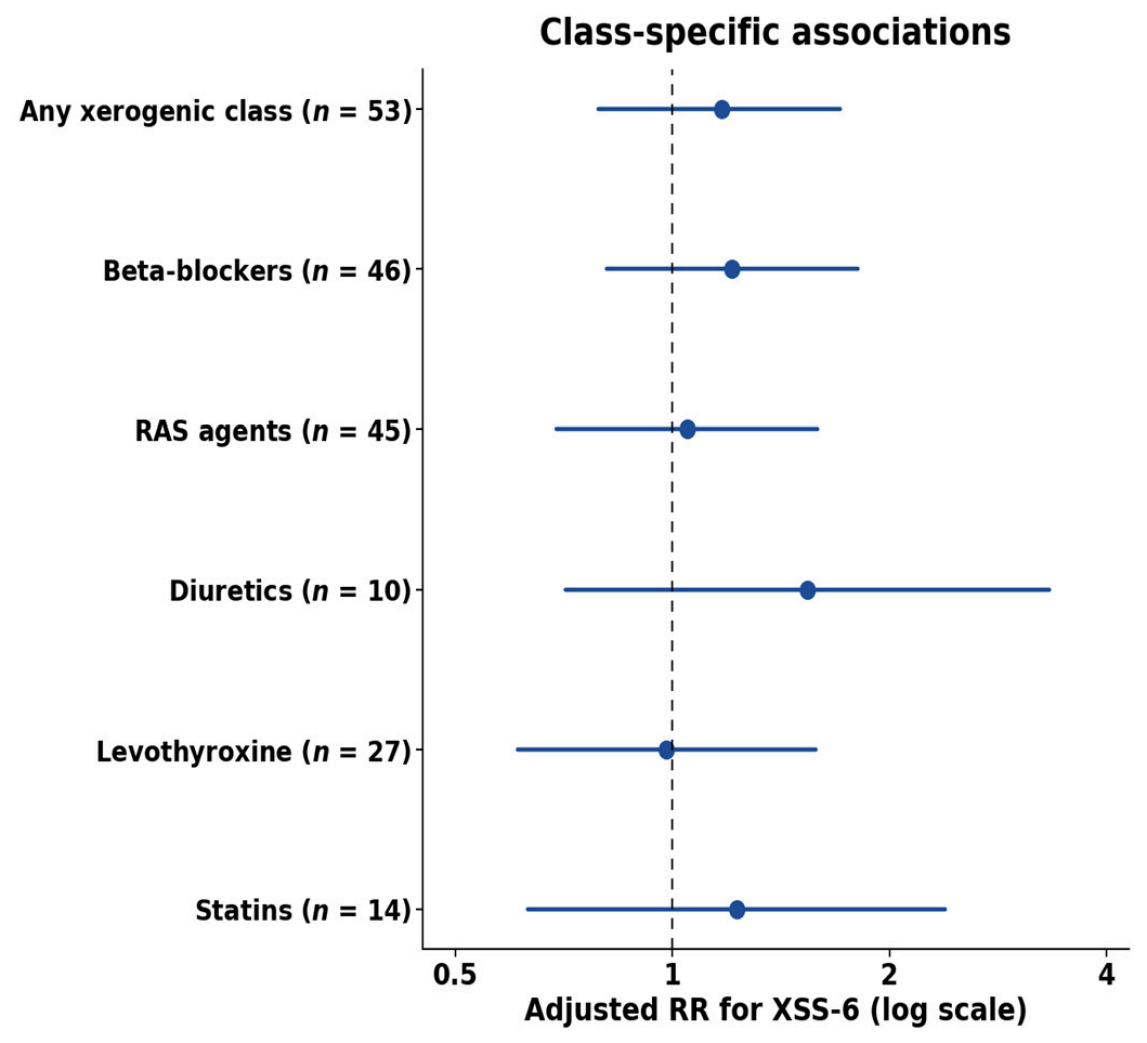
Adjusted associations between selected therapeutic classes and XSS-6 xerostomia burden. Points represent adjusted rate ratios (RRs) and horizontal bars indicate 95% confidence intervals from negative binomial regression models. The dashed line indicates the null value (RR = 1). Sample sizes correspond to complete cases included in each model. RAS, renin–angiotensin system; RR, rate ratio.

Secondary analyses examined whether the association with XSS-6 differed according to therapeutic class or overall medication burden. In class-specific negative binomial models adjusted for age, menopausal status, BMI, current smoking, higher medication burden, and PSY-9, none of the individual therapeutic classes evaluated was independently associated with XSS-6 score. The adjusted rate ratio was 1.21 (95% CI 0.81–1.81) for β-blockers, 1.05 (95% CI 0.69–1.59) for renin–angiotensin-system agents, 1.54 (95% CI 0.71–3.33) for diuretics, and 0.98 (95% CI 0.61–1.58) for levothyroxine (Figure 3). Confidence intervals were wide, particularly for less frequently used medication classes, and these analyses were considered exploratory.

Medication burden showed a positive but attenuated association with XSS-6 after multivariable adjustment. When the questionnaire-derived medication categories were modelled ordinally, the adjusted rate ratio was 1.21 per category increase (95% CI 1.00–1.46; *p* for trend = 0.051). Compared with women reporting no regular chronic medication or medication for acute conditions only, the corresponding rate ratios were 1.38 for those reporting 1–2 daily tablets and 1.39 for those reporting ≥3 daily tablets.

**Figure 4:**
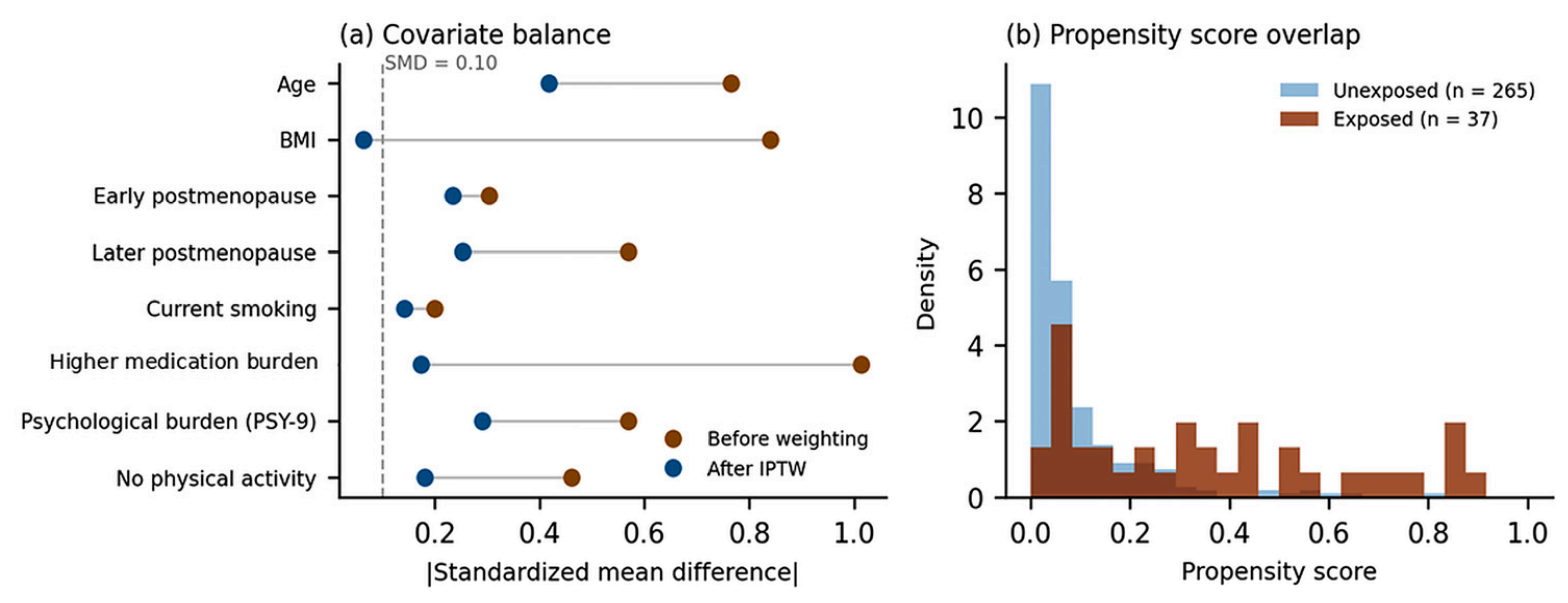
Propensity-score assessment according to participant-reported xerogenic medication exposure. (**a**) Absolute standardized mean differences for covariates before and after stabilized, truncated inverse-probability-of-treatment weighting; the dashed line indicates an absolute SMD of 0.10. (**b**) Propensity-score distributions among women with and without participant-reported xerogenic medication exposure. The analysis included 302 participants with complete covariate data. SMD, standardized mean difference.

Because women with and without participant-reported xerogenic medication exposure differed in several baseline characteristics, propensity-score weighting was used as a sensitivity analysis. Stabilized inverse-probability-of-treatment weighting reduced the absolute standardized mean differences for BMI from 0.84 to 0.06, higher medication burden from 1.01 to 0.17, physical inactivity from 0.46 to 0.18, and PSY-9 from 0.57 to 0.29. Residual imbalance remained, particularly for age (SMD 0.77 before vs. 0.42 after weighting), indicating that weighting did not achieve complete covariate balance (Figure 4a). Propensity-score distributions showed overlap between the exposure groups (Figure 4b).

Given the residual imbalance after weighting, the weighted outcome models additionally adjusted for age, menopausal status, and PSY-9. In these models, participant-reported xerogenic medication exposure was associated with a higher complete XSS-6 score (aRR 1.81, 95% CI 1.19–2.73; *p* = 0.005) and greater XSS-6 severity among symptomatic women (adjusted difference, 1.70 points; 95% CI 0.45–2.95; *p* = 0.008). These estimates were directionally consistent with the primary regression analyses but were considered supportive sensitivity findings given the residual covariate imbalance and the relatively small exposed group.

Additional sensitivity analyses examined the robustness of the findings to alternative analytical specifications. Exclusion of the 19 women reporting menopausal hormone therapy use yielded an adjusted difference in XSS-6 severity of 1.23 points (95% CI 0.14–2.31) and an adjusted rate ratio of 1.58 (95% CI 1.01–2.48) for the complete XSS-6 score. A zero-inflated negative binomial model yielded an exposure rate ratio of 1.45 (95% CI 1.10–1.91; *p* = 0.009) in its count component.

When the medication-list-derived definition replaced participant-reported exposure in the full nested modelling strategy, unadjusted and demographically adjusted associations with XSS-6 were evident (M0: any-symptom OR 4.00, 95% CI 1.53–10.43; rate ratio 1.50, 95% CI 1.08–2.10; M1: OR 3.27, 95% CI 1.22–8.80; rate ratio 1.47, 95% CI 1.03–2.10), but estimates attenuated substantially after additional adjustment for medication burden and psychological symptom burden (M2: OR 2.02, 95% CI 0.67–6.08; severity difference 0.06 points, 95% CI −0.85 to 0.96; rate ratio 1.17, 95% CI 0.79–1.71) (Table 7). Because this exposure definition overlaps with overall medication burden, adjustment for the number of daily medicines in M2 may partly adjust away the pharmacological exposure itself, and the M1 estimates may better reflect the pharmacological association. For the OSS-9 outcome, the fully adjusted odds ratio for any finding was 2.52 (95% CI 1.00–6.35). These results indicate that the two exposure definitions captured related but non-equivalent constructs and that associations based on participant-reported exposure should be interpreted as an upper bound.

### 2.6. Concordance Between Medication-List-Derived and Participant-Reported Xerogenic Exposure

Medication-list–derived and participant-reported xerogenic exposure showed 85.3% overall agreement, with moderate agreement beyond chance (Cohen’s κ = 0.43). Among the 54 women whose medication listings included at least one therapeutic class classified as having recognized xerogenic potential, 24 (44.4%) also reported taking medication capable of causing dry mouth.

Within this medication-list–identified subgroup, participant recognition of xerogenic medication exposure was associated with higher XSS-6 scores (OR 1.41 per XSS-6 point, 95% CI 1.07–1.86) and higher medication burden (OR 4.71, 95% CI 1.21–18.31). Given the cross-sectional design, these associations indicate that recognition differed according to xerostomia symptom burden and medication burden but do not establish the temporal relationship between symptom development and awareness of potential xerogenic medication effects. Accordingly, participant recognition was interpreted throughout as a drug-safety awareness measure rather than as evidence of underreporting to pharmacovigilance systems.

## 3. Discussion

In this cross-sectional cohort of 306 midlife Romanian women, participant-reported use of medication potentially linked with dry mouth was associated with a near-universal prevalence of xerostomia symptoms (94.9%) and with an adjusted excess of approximately 1.3 points on the 18-point XSS-6 score among symptomatic women, independent of age, menopausal stage, BMI, smoking, medication burden, and psychological burden. The magnitude of the association decreased after full adjustment, particularly after accounting for psychological and medication burden, but remained evident for symptom severity. This pattern is consistent with recent multicentre evidence linking systemic medication exposure and polypharmacy with impaired salivary flow, including among users of antidepressant, antihypertensive, benzodiazepine, and diuretic therapies [26]. In contrast, when exposure was defined pharmacologically from the medication listings, the fully adjusted estimates were close to the null (Table 7), so the participant-reported associations are best read as an upper bound that may incorporate symptom-driven recognition of medication effects.

Complementary analyses supported the direction of the primary association: the zero-inflated count model yielded a rate ratio of 1.45, propensity-score-weighted analysis a rate ratio of 1.81, and exclusion of MHT users produced comparable estimates. These results should nevertheless be interpreted as convergent sensitivity evidence rather than proof of causality, particularly given the residual imbalance after propensity-score weighting. Furthermore, medication exposure was not independently associated with the broader OSS-9 outcome. This divergence suggests that the observed medication-related signal was more specific to xerostomia burden than to general oral-health complaints. Such separation is clinically relevant because xerostomia, salivary dysfunction, and broader oral health-related quality of life are related but non-equivalent constructs. In a large 2025 study of 3211 peri- and postmenopausal women, xerostomia was highly prevalent and associated with poorer oral health-related quality of life, whereas menopausal status itself was not independently associated with xerostomia. Similarly, among 327 postmenopausal women, menopausal duration was associated with salivary flow but not with oral health-related quality of life [27,28].

These findings extend evidence linking pharmacotherapy with subjective oral dryness in older and general adult populations [6,7,8] to women traversing the menopausal transition. In a 2021 study of perimenopausal women aged 45–55 years, xerostomia was associated with the total number of medications, psychotropic medication use, age, and climacteric symptoms, illustrating how pharmacological and menopause-related factors may coexist in this age group [29].

The high unadjusted odds ratio observed in our study (7.4) should therefore not be interpreted as indicating an unusually large pharmacological effect. Only two participant-reported exposed women had no XSS-6 symptoms, and the association was substantially attenuated after multivariable adjustment. The unadjusted estimate probably captures the combined influence of medication burden, psychological symptoms, and the participant-reported exposure definition. Nevertheless, biological susceptibility during the menopausal transition remains plausible. Lower salivary estradiol concentrations have been reported in postmenopausal women with xerostomia, while other contemporary studies have documented reduced salivary flow and altered oral-health indices after menopause [30,31]. However, our study was not designed to demonstrate a pharmacological–hormonal interaction, and menopausal stage should be regarded primarily as part of the clinical context in which medication-associated dryness occurs.

The relationship with medication burden further supports this interpretation. Dry mouth has long been associated with the number of medicines taken rather than exclusively with individual agents [6,7]. In our cohort, the medication–count gradient was strong in unadjusted analyses but attenuated after full adjustment (*p*-trend = 0.051). Recent systematic and meta-analytic evidence similarly associates increasing medication burden and polypharmacy with dry mouth and hyposalivation, although most available evidence concerns older adults [32,33]. The questionnaire captured medication use in three structured categories (no regular chronic medication or acute use only, 1–2 daily tablets, and ≥3 daily tablets). These categories do not correspond to the conventional definition of polypharmacy (typically ≥5 concomitant medicines); the term medication burden is therefore used throughout, and the ≥3-tablet threshold reflects the available questionnaire categorization rather than a standard polypharmacy cut-off.

No individual therapeutic class reached statistical significance in our class-specific analyses, and several estimates had wide confidence intervals. Diuretics produced the largest point estimate, but this should not be interpreted as evidence of a class-specific effect; conversely, the near-null estimate for levothyroxine does not establish pharmacological neutrality. Medication-associated xerostomia can arise through multiple mechanisms, including effects on autonomic signalling, central regulation, fluid and electrolyte balance, and receptor-mediated salivary secretion [34]. Several classes, including psychotropic agents, antihistamines, antiepileptics, and centrally acting antihypertensives, were reported by very few participants, so the absence of statistically significant class-specific estimates must not be read as evidence of safety for any individual class, and xerogenic potential can vary with dose and agent within the same therapeutic class.

Cumulative pharmacological burden may therefore be more informative than the presence of an individual therapeutic class. A recent systematic review found associations between anticholinergic medication exposure, cumulative anticholinergic burden, and xerostomia, supporting assessment of the overall xerogenic medication profile rather than attribution to a single drug class [35]. Clinical studies of patients receiving xerogenic medications likewise demonstrate the importance of medication combinations and patient-level susceptibility [36].

An additional consideration is confounding by indication. The conditions for which these medicines are prescribed, including hypertension, diabetes, depression and anxiety, and autoimmune disease, can themselves influence oral dryness through disease-related mechanisms, so part of the observed association may reflect the underlying illness rather than its treatment. Disentangling drug effects from indication effects would require designs with information on treatment initiation, indication, and disease severity.

Psychological burden was another important determinant of oral symptoms. PSY-9 remained strongly associated with XSS-6 and OSS-9 after adjustment, consistent with the established relationship between anxiety, depression, stress, and subjective oral dryness [20]. Contemporary clinical data similarly show associations between xerostomia and psychological symptoms, while studies in perimenopausal women link greater menopausal symptom burden with xerostomia and other oral sensory complaints [37,38].

The attenuation of the medication estimate after inclusion of PSY-9 is therefore clinically informative rather than simply a loss of statistical strength. A further pharmacological overlap should be noted: women with higher PSY-9 scores are also more likely to be prescribed psychotropic medicines with established xerogenic potential (for example SSRIs and benzodiazepine-related agents). Adjustment for psychological symptom burden may therefore absorb part of the pharmacological effect of the drugs used to treat that burden, in addition to capturing non-pharmacological psychogenic contributions to perceived dryness. Psychological burden is an important covariate when evaluating suspected medication-associated xerostomia, and recent evidence across the menopausal transition also links depressive symptom burden with poorer oral health-related quality of life [39]. Clinically, persistent dry-mouth complaints should not be attributed automatically either to pharmacotherapy or to psychological symptoms; both warrant assessment.

Menopausal stage showed a different pattern: it was not independently associated with XSS-6 severity, whereas later postmenopausal status was associated with the occurrence of broader OSS-9 findings. This is compatible with evidence that menopause-related oral changes extend beyond xerostomia to altered salivary characteristics, periodontal disease, tooth loss, taste disturbances, and mucosal disorders. A recent scoping review also emphasized the contribution of age, medication exposure, and treatment for climacteric symptoms, supporting a multifactorial rather than exclusively hormonal interpretation [40]. Accordingly, our findings support considering menopausal status as part of the clinical background against which medication-associated dryness develops rather than as a sufficient explanation for xerostomia. Only 6.2% of participants used MHT, a level consistent with the low uptake reported in Romania and much of Eastern Europe but well below treatment rates described in Western European and North American surveys. The present findings may therefore not generalize directly to populations in which MHT use is common and hormonal treatment could modify both oral symptoms and medication profiles.

From a drug-safety and risk-management perspective, three findings deserve emphasis. First, xerostomia is a predictable and potentially modifiable adverse effect. Medication review, patient education, oral-hygiene measures, saliva substitutes, and pharmacological stimulation where appropriate remain central components of management [41,42,43].

Second, medication-associated oral adverse effects should not be considered exclusively a geriatric problem. Nearly half of our midlife cohort used chronic medication, with antihypertensives predominating. Medication-induced xerostomia and impaired salivary function have also been documented specifically in middle-aged adults receiving long-term xerogenic or anticholinergic medications, while greater anticholinergic burden has been associated with poorer dental status [44,45]. Recent evidence in postmenopausal women further illustrates the complexity of this relationship: SSRI users had lower stimulated salivary flow than HRT users or untreated controls, whereas subjective xerostomia did not parallel salivary-flow differences. This discordance reinforces the distinction between subjective xerostomia and objectively measured hyposalivation [46].

Third, the discrepancy between medication-list-derived and participant-reported xerogenic exposure represents a potentially important drug-safety finding. Among 54 women whose medication lists contained at least one recognized xerogenic class, only 24 (44.4%) reported taking a medication capable of causing dry mouth. Recognition was more likely among women with greater XSS-6 symptom burden and higher medication burden. Because the study is cross-sectional, we cannot determine whether symptoms prompted recognition, previous counselling increased awareness, or another mechanism explains this association. Nevertheless, the finding suggests a potential communication gap. Contemporary pharmacovigilance research similarly identifies knowledge, beliefs, healthcare context, and professional support as important determinants of patient recognition and reporting of suspected adverse drug reactions [47]. Recent reviews further identify limited knowledge, complex reporting processes, inadequate feedback, and insufficient professional support as barriers to patient participation in pharmacovigilance [48]. Comparable gaps exist among healthcare professionals, for whom uncertainty regarding what, when, and how to report suspected adverse reactions remains an important barrier [49]. These barriers are potentially modifiable: educational interventions have been shown to increase adverse-drug-reaction reporting among healthcare professionals [50].

Our questionnaire assessed recognition rather than formal ADR reporting; the 44.4% recognition rate therefore describes a medication-safety awareness and communication gap and is not evidence of underreporting to pharmacovigilance systems. Proactive counselling about xerogenic effects and multidisciplinary medication review may therefore represent pragmatic risk-minimization measures, particularly for women receiving multiple potentially xerogenic medicines.

Topical management and saliva substitutes can provide symptomatic relief [42,43], while caries prophylaxis is warranted in persistent hyposalivation [4,51]. However, symptomatic treatment should complement rather than replace assessment of potentially modifiable medication exposure. Where clinically feasible, medication review, assessment of cumulative xerogenic burden, dose optimization, and consideration of less xerogenic alternatives may address pharmacological contributors while preventive dental measures reduce downstream complications [52]. Persistent xerostomia and salivary hypofunction increase susceptibility to dental and mucosal complications, further supporting integration of medication review with preventive oral care [53].

Strengths of this study include the prespecified nested adjustment strategy, triangulation across two-part, zero-inflated, and propensity-score-weighted specifications, complementary exposure definitions, and transparent reporting of analyses that attenuated or did not support the primary association. The parallel evaluation of XSS-6 and OSS-9 also allowed medication-associated xerostomia burden to be distinguished from broader oral-health symptoms.

But several limitations should be considered. The cross-sectional design precludes causal or temporal inference. Both medication exposure and outcomes were participant-reported, and salivary flow was not objectively measured. The XSS-6 and OSS-9 were derived from MMBQ-44 items that mirror, but are not identical to, the validated XI and OHIP instruments, limiting direct comparison with studies using the complete scales. Moreover, subjective xerostomia and objectively measured hyposalivation are not interchangeable, reinforcing the value of sialometry in future studies. For the same reason, the present results concern the subjective sensation of dry mouth and not objectively measured hyposalivation, and the two terms are used accordingly throughout.

Furthermore, the participant-reported exposure item explicitly referred to medications capable of causing dry mouth and may therefore have been influenced by existing symptoms. The attenuation observed with the medication-list-derived definition, together with the association between symptom burden and recognition of xerogenic medication use, is consistent with possible outcome-dependent exposure classification. Consequently, the stronger association obtained with participant-reported exposure should not be interpreted as a direct estimate of a pharmacological effect. Conversely, the medication-list-derived definition was limited by therapeutic-class coding and the absence of information on medication dose, duration, adherence, and indication. In particular, no data were available on dose, treatment duration, adherence, indication, or the timing of symptom onset relative to treatment initiation, and cumulative anticholinergic load could not be quantified with a validated scale; such measures could be more informative than the presence of a single agent from a xerogenic class.

Finally, the participant-reported exposed group was small (n = 39), producing wide confidence intervals for some estimates and limited power for class-specific analyses. Residual age imbalance remained after propensity-score weighting, sensitivity analyses were exploratory and not adjusted for multiplicity, and residual confounding by comorbidity, hydration, specific drug combinations, anticholinergic burden, dental status, or other determinants of salivary function cannot be excluded. Community-based voluntary recruitment and the Romanian setting may also limit generalizability to populations with different prescribing patterns, healthcare access, or sociodemographic characteristics.

Notwithstanding these limitations, the convergence of findings across complementary analytical approaches provides a coherent basis for considering medication exposure alongside menopausal, psychological, and oral-health factors when interpreting xerostomia in midlife women.

## 4. Materials and Methods

### 4.1. Study Design and Setting

This cross-sectional, questionnaire-based study included Romanian women aged 45–65 years and was conducted between December 2025 and July 2026 within a university-affiliated observational research project. Participants were recruited voluntarily from the community, independently of oral-health status, dental-care attendance, or gynaecological consultation.

The present analysis used data from the cohort in which the Multidimensional Menopausal Burden Questionnaire (MMBQ-44) was previously psychometrically validated [23]. The English version of the questionnaire used in the study is provided as Supplementary File S1. The previous study focused on the psychometric validation of the MMBQ-44 [23]; the present analysis addresses a distinct drug-safety question concerning medication exposure and oral symptom burden.

The study was conducted in accordance with the Declaration of Helsinki and was approved by the Ethics Committee of Victor Babeș University of Medicine and Pharmacy, Timișoara (approval No. 75/28.11.2025). All participants provided written informed consent before completing the questionnaire. Participation was voluntary, and questionnaire responses were collected anonymously.

### 4.2. Participants and Menopausal Classification

A total of 308 questionnaires were received. Two respondents did not provide informed consent and were excluded, resulting in a final analytical sample of 306 women.

Menopausal status was derived from participant-reported time since the final menstrual period (FMP), as recorded in the questionnaire. For the present analyses, participants were grouped into three categories: perimenopausal (n = 65), early postmenopausal (n = 40), and later postmenopausal (n = 201). These categories were used as pragmatic analytical groupings to examine medication-associated xerostomia and oral symptom burden across different phases of the menopausal transition.

### 4.3. Medication Exposure Assessment

Medication exposure was assessed using complementary information collected through the MMBQ-44, including current medication use, free-text reporting of medication names, the reported number of medications taken regularly, and participant-reported use of medications potentially associated with dry mouth [23].

Two prespecified exposure definitions were used and evaluated with the same nested modelling strategy. The medication-list-derived definition, based on therapeutic-class coding of the reported medicines, was regarded as the pharmacological reference exposure; participant-reported use of medication potentially associated with dry mouth was analysed in parallel and additionally interpreted as an indicator of patient recognition of xerogenic exposure.

The participant-reported exposure was derived from the questionnaire item asking whether the participant was taking medication that could cause a sensation of dry mouth, with antihypertensive and antidepressant medications provided as examples. An affirmative response was recorded for 39 participants (12.7%). Because the item names dry mouth explicitly, this measure combines pharmacological exposure with awareness of the potential adverse effect and may be influenced by existing symptoms.

For the medication-list-derived definition, medications reported in the free-text field were coded according to therapeutic class. Participants reporting at least one medication belonging to a prespecified class with recognized xerogenic potential were classified as medication-list exposed. The classification included diuretics, including relevant fixed-dose combinations; β-blockers; centrally acting antihypertensive agents; selective serotonin reuptake inhibitors and benzodiazepine-related agents; antihistamines; and antiepileptic medications [1,8]. Based on this definition, 54 participants (17.6%) were classified as exposed to at least one medication from a recognized xerogenic class. Agreement between the participant-reported and medication-list-derived exposure definitions was assessed using percentage agreement and Cohen’s κ. The full mapping between reported medication names, assigned therapeutic classes, xerogenic classification, and the criterion used for classification [1,8] is provided in Supplementary Table S1.

Overall medication use and medication burden were characterized separately. Medication burden was based on the structured questionnaire categories distinguishing no regular medication for a chronic condition or medication used only for acute conditions, 1–2 daily tablets, and ≥3 daily tablets. Injectable treatment was described separately. Because these questionnaire categories do not correspond to a conventional definition of polypharmacy, the term “medication burden” was used throughout the present analysis.

### 4.4. Xerostomia and Oral Symptom Outcomes

The primary outcome was xerostomia symptom burden, assessed using a six-item score (XSS-6; range 0–18) derived from the oral-symptom component of the MMBQ-44 [23]. The six items assessed dry-mouth sensation, difficulty swallowing dry foods, the need to sip liquids to facilitate swallowing, difficulty speaking, dry or cracked lips, and nocturnal thirst. These items conceptually reflect established Xerostomia Inventory content [17,18]. Each item was scored from 0 (never) to 3 (always), and item scores were summed, with higher total scores indicating greater xerostomia symptom burden.

For a complementary binary analysis, higher xerostomia symptom burden was defined as XSS-6 ≥6. This threshold was specified as an exploratory, distribution-based cutoff corresponding approximately to the upper quintile of the observed XSS-6 distribution. It was used for analytical purposes and was not interpreted as a validated diagnostic or clinical threshold.

The secondary outcome was broader oral symptom burden, assessed using a nine-item oral symptom score (OSS-9; range 0–27) derived from the oral-health component of the MMBQ-44 [23]. The items reflected oral-health impact constructs [19] and assessed dental problems affecting pronunciation, gingival bleeding, white or red oral mucosal lesions, oral ulcerations, previous oral candidiasis, white coating of the tongue or buccal mucosa, diagnosed periodontal problems, embarrassment related to teeth or dentures, and interference of oral problems with daily activities. Each item was scored from 0 (never) to 3 (always), with higher total scores indicating greater oral symptom burden. Internal consistency in the present sample was acceptable for both derived scores (XSS-6: Cronbach’s α = 0.76; OSS-9: Cronbach’s α = 0.82), comparable to the previously reported internal consistency of the PSY-9 subscale (Cronbach’s α = 0.94).

Smell and taste alterations were summarized separately using the corresponding questionnaire items and were treated as descriptive secondary outcomes rather than components of the primary xerostomia or oral symptom scores.

### 4.5. Covariates

Potential covariates were selected a priori on the basis of their plausible relationships with medication exposure, xerostomia, oral symptoms, or menopausal symptom burden. The principal covariates were age, body mass index (BMI), menopausal status, current smoking, medication burden, and psychological symptom burden. Higher medication burden was defined as the use of ≥3 daily tablets and was included as a binary covariate in the principal multivariable models.

BMI was calculated from participant-reported height and weight. Psychological symptom burden was assessed using the nine-item psychological component of the MMBQ-44 (PSY-9), with each item scored from 0 to 4 and summed to provide a total score ranging from 0 to 36; higher scores indicate greater psychological symptom burden. Internal consistency of the PSY-9 in the present sample was high (Cronbach’s α = 0.94) [23].

Educational attainment, alcohol consumption, physical activity, and menopausal hormone therapy (MHT) use were additionally described. These variables were considered in sensitivity or supplementary analyses, where appropriate, rather than routinely included in the principal multivariable models, to maintain model parsimony relative to the sample size and the number of participants reporting xerogenic medication exposure.

### 4.6. Statistical Analysis

Because both outcome scores were semicontinuous with a point mass at zero, the primary analysis used two-part (hurdle) models [54,55]. Part 1 modelled the probability of reporting any symptoms (score > 0) using logistic regression, whereas Part 2 modelled symptom severity among women with scores > 0 using linear regression with heteroscedasticity-robust (HC3) standard errors. Three prespecified nested adjustment sets were fitted: M0 (exposure only), M1 (M0 plus age, menopausal stage, BMI, and current smoking), and M2 (M1 plus higher medication burden [≥3 daily tablets] and PSY-9 psychological symptom burden). Negative binomial regression applied to the complete outcome score was used as a complementary count-model specification.

Secondary and sensitivity analyses were conducted to assess the consistency of the primary findings. Class-specific negative binomial models estimated adjusted rate ratios for the therapeutic classes sufficiently represented in the medication listings, including β-blockers, renin–angiotensin-system agents, diuretics, levothyroxine, and lipid-lowering agents. Medication burden was additionally examined using the questionnaire-derived categories (no regular chronic medication or medication for acute conditions only, 1–2 daily tablets, and ≥3 daily tablets), both categorically and as an ordinal term to assess evidence of an exposure–response pattern.

To address baseline differences between women with and without participant-reported xerogenic medication exposure, a propensity score was estimated from age, BMI, menopausal stage, current smoking, higher medication burden, PSY-9, and physical inactivity [42]. Stabilized inverse-probability-of-treatment weights were truncated at the 99th percentile [56], and covariate balance before and after weighting was evaluated using absolute standardized mean differences (SMDs). Because residual imbalance remained after weighting, weighted outcome models additionally adjusted for age, menopausal stage, and PSY-9.

Additional sensitivity analyses included a zero-inflated negative binomial model [43], exclusion of women reporting current menopausal hormone therapy use, and replacement of participant-reported xerogenic medication exposure with the medication-list-derived definition based on therapeutic classes with recognized xerogenic potential [1,8]. Agreement between participant-reported and medication-list-derived xerogenic exposure was quantified using percentage agreement and Cohen’s κ. Among women classified as exposed according to their medication listings, exploratory logistic regression was used to examine whether participant recognition of xerogenic medication exposure was associated with XSS-6 symptom burden and higher medication burden. The medication-list-derived exposure was evaluated with the same nested M0–M2 specification as the participant-reported measure, and those results are reported in full (Table 7).

Group comparisons used the Mann–Whitney U test for continuous variables and the χ^2^ or Fisher’s exact test for categorical variables, as appropriate. Multicollinearity was assessed using variance inflation factors (all <2.0). Analyses used complete cases (≥98.7% of the sample per primary model), and all tests were two-sided with α = 0.05. Secondary and sensitivity analyses were considered supportive and exploratory; no adjustment for multiplicity was applied, and their interpretation emphasized effect estimates, confidence intervals, and consistency across analytical specifications rather than isolated statistical significance. Analyses were performed using JASP (version 0.19.3; JASP Team, Amsterdam, The Netherlands).

## 5. Conclusions

The present findings identify xerostomia as a relevant oral symptom burden in midlife women. The association with medication exposure depended on how exposure was defined: participant-recognized xerogenic medication use remained associated with greater xerostomia severity after full adjustment, whereas the pharmacologically defined, medication-list-derived exposure showed unadjusted associations that attenuated to near-null estimates in fully adjusted models. Psychological symptom burden was the most consistent independent correlate of both oral outcomes. The signal was specific to xerostomia rather than to broader oral symptoms, but the cross-sectional design, participant-reported measures, and the dependence of the estimates on the exposure definition preclude causal attribution.

The finding that fewer than half of women whose medication lists included a recognized xerogenic class identified themselves as taking medication capable of causing dry mouth points to a medication-safety awareness and communication gap rather than to underreporting of adverse reactions. These outcomes support greater attention to xerostomia during medication review and counselling in women across the menopausal transition, particularly in the presence of higher medication and psychological symptom burden. Prospective studies integrating verified medication exposure, dose and duration data, validated xerostomia instruments, and objective salivary-flow measurements are needed to establish temporality and determine whether reducing xerogenic medication burden or implementing structured medication counselling can prevent or alleviate symptoms.

## Figures and Tables

**Table 1:** Participant characteristics according to participant-reported xerogenic medication exposure (n = 306).

Characteristic	Unexposed (n = 267)	Exposed (n = 39)	*p* ^1^
Age, years	52.9 ± 5.4	56.8 ± 5.0	<0.001
BMI, kg/m^2^	26.8 ± 4.8	31.4 ± 6.8	<0.001
Menopausal stage, n (%)			0.028
Perimenopausal	61 (22.8)	4 (10.3)	
Early postmenopausal	38 (14.2)	2 (5.1)	
Later postmenopausal	168 (62.9)	33 (84.6)	
University education, n (%)	189 (71.3)	23 (59.0)	0.168
Current daily smoking, n (%)	47 (17.6)	4 (10.3)	0.358
Alcohol consumption, n (%)	62 (23.4)	8 (20.5)	0.845
No regular physical activity, n (%)	47 (17.6)	14 (35.9)	0.014
Current MHT use, n (%)	17 (6.4)	2 (5.1)	1.000
Higher medication burden (≥3 daily tablets), n (%)	36 (13.6)	21 (56.8)	<0.001
Psychological burden, PSY-9 (0–36)	9.9 ± 8.3	14.8 ± 9.6	0.002
Vasomotor score (0–8)	2.3 ± 2.4	3.0 ± 2.6	0.113

^1^ Mann–Whitney U test (continuous variables, mean ± SD) or χ^2^/Fisher’s exact test (categorical variables). Denominators vary slightly for education, alcohol, and medication burden because of missing responses. BMI, body mass index; MHT, menopausal hormone therapy.

**Table 2:** Therapeutic classes identified from free-text medication listings in the study population (n = 306; chronic medication users, n = 131).

Therapeutic Class	n (% of Total Sample; n = 306)	n (% of Chronic Medication Users; n = 131)
Renin–angiotensin-system agents (ACE inhibitors/ARBs, including combinations)	47 (15.4)	47 (35.9)
β-blockers ^1^	47 (15.4)	47 (35.9)
Levothyroxine	28 (9.2)	28 (21.4)
Statins/lipid-lowering agents	15 (4.9)	15 (11.5)
Antiplatelet agents	13 (4.2)	13 (9.9)
Diuretics, including fixed-dose combinations ^1^	10 (3.3)	10 (7.6)
Calcium-channel blockers	9 (2.9)	9 (6.9)
Venoactive agents	9 (2.9)	9 (6.9)
Antidiabetic agents	5 (1.6)	5 (3.8)
Osteoporosis therapy	5 (1.6)	5 (3.8)
Psychotropics (SSRIs and benzodiazepine-related agents) ^1^	3 (1.0)	3 (2.3)
Antihistamines ^1^	2 (0.7)	2 (1.5)
Antiepileptics ^1^	2 (0.7)	2 (1.5)
Centrally acting antihypertensives ^1^	1 (0.3)	1 (0.8)
Other medications ^2^	11 (3.6)	11 (8.4)

^1^ Therapeutic class considered to have recognized xerogenic potential according to the prespecified medication classification [1,8] and included in the medication-list-derived xerogenic exposure definition. Overall, 54 participants (17.6% of the total sample) reported at least one medication belonging to a class classified as having recognized xerogenic potential. ^2^ Includes less frequently reported medications, such as allopurinol and agents used for migraine prophylaxis. Therapeutic classes are not mutually exclusive because participants could report medications from more than one class. ACE, angiotensin-converting enzyme; ARB, angiotensin II receptor blocker; SSRI, selective serotonin reuptake inhibitor.

**Table 3:** Xerostomia and oral symptom outcomes according to participant-reported xerogenic medication exposure (n = 306).

Outcome	No Participant-Reported Exposure (n = 267)	Participant-Reported Exposure (n = 39)	*p* ^1^
Any XSS-6 symptom, n (%)	191 (71.5)	37 (94.9)	0.001
XSS-6 score, mean ± SD	2.6 ± 2.7	4.8 ± 3.1	<0.001
XSS-6 score, median (IQR)	2 (0–4)	4 (2–7)	—
Higher xerostomia burden (XSS-6 ≥ 6), n (%)	45 (16.9)	13 (33.3)	0.026
Any OSS-9 finding, n (%)	162 (60.7)	30 (76.9)	0.053
OSS-9 score, mean ± SD	2.6 ± 3.4	3.6 ± 3.7	0.024
OSS-9 score, median (IQR)	1 (0–4)	3 (1–5)	—
Chemosensory score (0–6), mean ± SD	0.7 ± 1.2	1.3 ± 1.7	0.048

^1^ Fisher’s exact test or χ^2^ test for categorical variables and Mann–Whitney U test for continuous scores, as appropriate. XSS-6, six-item xerostomia symptom score; OSS-9, nine-item oral symptom score; IQR, interquartile range; SD, standard deviation.

**Table 4:** Association between participant-reported xerogenic medication exposure and XSS-6 xerostomia burden across nested adjustment models.

Model	Any Symptom, OR (95% CI)	*p*	Severity, Symptomatic Women, β (95% CI)	*p*	Complete Score, RR (95% CI)	*p*
M0 unadjusted	7.36 (1.73–31.30)	0.007	1.51 (0.48–2.54)	0.004	1.89 (1.30–2.74)	<0.001
M1 + age, stage, BMI, smoking	6.11 (1.40–26.66)	0.016	1.72 (0.65–2.78)	0.002	1.85 (1.24–2.76)	0.003
M2 + higher medication burden, PSY-9	4.18 (0.81–21.62)	0.088	1.28 (0.22–2.34)	0.018	1.51 (0.97–2.34)	0.066

Part 1 used logistic regression for the presence of any XSS-6 symptom (score >0; n = 306 in M0/M1 and n = 302 in M2). Part 2 used linear regression with HC3 robust standard errors among symptomatic women (n = 228 in M0/M1 and n = 224 in M2); β represents the difference in XSS-6 points associated with participant-reported xerogenic medication exposure. Negative binomial regression was applied to the complete XSS-6 score. M0, unadjusted; M1, adjusted for age, menopausal status, BMI, and current smoking; M2, additionally adjusted for higher medication burden (≥3 daily tablets) and PSY-9 psychological symptom burden. The “Any symptom” columns report logistic-regression odds ratios; the “Severity” columns report linear-regression differences in XSS-6 points among symptomatic women; the “Complete score” columns report negative-binomial rate ratios. These estimands are not interchangeable. OR, odds ratio; RR, rate ratio; CI, confidence interval; BMI, body mass index; PSY-9, nine-item psychological symptom burden score.

**Table 5:** Fully adjusted two-part model for the association between participant-reported xerogenic medication exposure and XSS-6 xerostomia burden.

Covariate	Any Symptom, OR (95% CI)	*p*	Severity, Symptomatic Women, β (95% CI)	*p*
Participant-reported xerogenic medication exposure	4.18 (0.81–21.62)	0.088	1.28 (0.22 to 2.34)	0.018
Age (per year)	1.01 (0.94–1.08)	0.879	−0.03 (−0.10 to 0.03)	0.330
Early postmenopause (vs. perimenopausal)	1.56 (0.58–4.20)	0.380	0.11 (−1.19 to 1.42)	0.864
Later postmenopause (vs. perimenopausal)	2.20 (0.94–5.17)	0.071	0.04 (−1.02 to 1.10)	0.945
BMI (per kg/m^2^)	1.05 (0.98–1.12)	0.160	−0.01 (−0.07 to 0.05)	0.675
Current daily smoking	1.99 (0.85–4.68)	0.115	0.33 (−0.46 to 1.12)	0.417
Higher medication burden (≥3 daily tablets)	0.86 (0.35–2.09)	0.733	0.16 (−0.84 to 1.16)	0.760
Psychological burden, PSY-9 (per point)	1.18 (1.12–1.25)	<0.001	0.15 (0.11 to 0.19)	<0.001

Part 1 used logistic regression for the presence of any XSS-6 symptom (n = 302; McFadden pseudo-R^2^ = 0.218). Part 2 used linear regression with HC3 robust standard errors among symptomatic women (n = 224; R^2^ = 0.304). OR, odds ratio; CI, confidence interval; BMI, body mass index; PSY-9, nine-item psychological symptom burden score.

**Table 6:** Association between participant-reported xerogenic medication exposure and OSS-9 oral symptom burden across nested adjustment models.

Model	Any Finding, OR (95% CI)	*p*	Severity, Women with Findings, β (95% CI)	*p*	Complete Score, RR (95% CI)	*p*
M0 unadjusted	2.16 (0.99–4.73)	0.054	0.47 (−0.92 to 1.87)	0.506	1.41 (0.96–2.06)	0.078
M1 + age, stage, BMI, smoking	1.60 (0.70–3.68)	0.268	0.25 (−1.16 to 1.66)	0.729	1.20 (0.80–1.81)	0.378
M2 + higher medication burden, PSY-9	1.14 (0.42–3.10)	0.804	−0.42 (−2.08 to 1.25)	0.623	1.05 (0.67–1.64)	0.831

Part 1 used logistic regression for the presence of any OSS-9 finding (n = 306 in M0/M1 and n = 302 in M2). Part 2 used linear regression with HC3 robust standard errors among women reporting at least one OSS-9 finding (n = 192 in M0/M1 and n = 190 in M2); β represents the difference in OSS-9 points associated with participant-reported xerogenic medication exposure. Negative binomial regression was applied to the complete OSS-9 score. M0, unadjusted; M1, adjusted for age, menopausal status, BMI, and current smoking; M2, additionally adjusted for higher medication burden (≥3 daily tablets) and PSY-9 psychological symptom burden. OR, odds ratio; RR, rate ratio; CI, confidence interval; BMI, body mass index; PSY-9, nine-item psychological symptom burden score.

**Table 7:** Association between medication-list-derived xerogenic exposure and XSS-6 xerostomia burden across nested adjustment models.

Model	Any Symptom, OR (95% CI)	*p*	Severity, Symptomatic Women, β (95% CI)	*p*	Complete Score, RR (95% CI)	*p*
M0 unadjusted	4.00 (1.53–10.43)	0.005	0.65 (−0.15 to 1.45)	0.109	1.50 (1.08–2.10)	0.016
M1 + age, stage, BMI, smoking	3.27 (1.22–8.80)	0.019	0.76 (−0.04 to 1.56)	0.062	1.47 (1.03–2.10)	0.032
M2 + higher medication burden, PSY-9	2.02 (0.67–6.08)	0.209	0.06 (−0.85 to 0.96)	0.900	1.17 (0.79–1.71)	0.436

Model specifications as in Table 4, with medication-list-derived exposure to at least one recognized xerogenic therapeutic class (n = 54) replacing participant-reported exposure. Part 1: logistic regression for any XSS-6 symptom (n = 306 in M0/M1; n = 302 in M2). Part 2: linear regression with HC3 robust standard errors among symptomatic women (n = 228 in M0/M1; n = 224 in M2). Negative binomial regression was applied to the complete XSS-6 score. OR, odds ratio; RR, rate ratio; CI, confidence interval.

## Data Availability

The original contributions presented in this study are included in the article/Supplementary Materials. Further inquiries can be directed to the corresponding authors.

## Supplementary Materials

The following supporting information can be downloaded at https://www.mdpi.com/article/10.3390/ph19101577/s1, Supplementary File S1: English version of the Multidimensional Menopausal Burden Questionnaire (MMBQ-44) used in the study; Supplementary Table S1: Reported medication names with assigned therapeutic classes, xerogenic classification, and classification criterion.

## Abbreviations

The following abbreviations are used in this manuscript:

ACEi	Angiotensin-converting-enzyme inhibitor
ADR	Adverse drug reaction
ARB	Angiotensin receptor blocker
AUC	Area under the receiver operating characteristic curve
BMI	Body mass index
CI	Confidence interval
FMP	Final menstrual period
IPTW	Inverse probability of treatment weighting
MHT	Menopausal hormone therapy
MMBQ-44	44-item Multidimensional Menopausal Burden Questionnaire
OHIP	Oral Health Impact Profile
OSS-9	Nine-item oral symptom score (derived from the MMBQ-44)
OR	Odds ratio
PSY-9	Nine-item psychological burden score of the MMBQ-44
RR	Rate ratio
SMD	Standardized mean difference
SSRI	Selective serotonin reuptake inhibitor
STROBE	Strengthening the Reporting of Observational Studies in Epidemiology
XSS-6	Six-item xerostomia symptom score (derived from the MMBQ-44; not the validated Xerostomia Inventory)
ZINB	Zero-inflated negative binomial
