# Supplementary material for: Medication-Associated Xerostomia and Oral Symptom Burden in Midlife Women: A Drug Safety Perspective Across the Menopausal Transition
Source: Pharmaceuticals (Basel). 2026 Oct 5;19(10):1577. doi: 10.3390/ph19101577 (PMC13708235; doi:10.3390/ph19101577)
Supplement: Supplementary file 1 [file pharmaceuticals-19-01577-s001.zip › Supplementary Table S1.pdf]

Supplementary Table S1. Reported medication names, assigned therapeutic classes, and xerogenic classification.

Medication names are reproduced exactly as written by participants in the free-text field, including spelling variants and brand names. Classes marked "Yes" contribute to the medication-list-derived xerogenic exposure definition; the classification criterion is the systematic review of medications inducing salivary gland dysfunction by Wolff et al. (2017) [1] together with the meta-analysis of dry-mouth risk by therapeutic class by Tan et al. (2018) [8]. Entries that could not be assigned to a therapeutic class (dietary supplements, vitamins, nonspecific descriptions) did not contribute to any exposure definition.

Reported name (as written)	n	Assigned therapeutic class	Xerogenic class
**concor**	16	Beta-blockers	Yes
**nebilet**	16	Beta-blockers	Yes
**eutirox**	12	Levothyroxine	No
**noliprel**	11	RAS agents (ACEi/ARB, incl. combinations)	No
**euthyrox**	9	Levothyroxine	No
**aspenter**	8	Antiplatelet agents	No
**atacand**	5	RAS agents (ACEi/ARB, incl. combinations)	No
**detralex**	5	Venoactive agents	No
**norvasc**	4	Calcium-channel blockers	No
**prestarium**	4	RAS agents (ACEi/ARB, incl. combinations)	No
**sortis**	4	Statins / lipid-lowering agents	No
**alfa d3**	3	Not classified (supplement/other)	No
**magneziu**	3	Not classified (supplement/other)	No
**tertensif**	3	Diuretics (incl. fixed-dose combinations)	Yes
**tertensiv**	3	Diuretics (incl. fixed-dose combinations)	Yes
**vesel due**	3	Venoactive agents	No
**vitamina d**	3	Not classified (supplement/other)	No
**zetovar**	3	Statins / lipid-lowering agents	No
**actonel**	2	Osteoporosis therapy	No

**alpha d3**	2	Not classified (supplement/other)	No
**amlessa**	2	RAS agents (ACEi/ARB, incl. combinations)	No
**antiinflamatoare**	2	Not classified (supplement/other)	No
**atacând**	2	RAS agents (ACEi/ARB, incl. combinations)	No
**betaloc**	2	Beta-blockers	Yes
**coaprovel**	2	RAS agents (ACEi/ARB, incl. combinations)	No
**euthirox**	2	Levothyroxine	No
**eutyrox**	2	Levothyroxine	No
**ibutin**	2	Other agents	No
**leridip**	2	Calcium-channel blockers	No
**milurit**	2	Other agents	No
**noliprel 2**	2	RAS agents (ACEi/ARB, incl. combinations)	No
**nu e cazu**	2	Not classified (supplement/other)	No
**procor**	2	Beta-blockers	Yes
**rosucard**	2	Statins / lipid-lowering agents	No
**siofor**	2	Antidiabetic agents	No
**triplixan**	2	RAS agents (ACEi/ARB, incl. combinations)	No
**tritace**	2	RAS agents (ACEi/ARB, incl. combinations)	No
**vit d**	2	Not classified (supplement/other)	No
**vitamina d3**	2	Not classified (supplement/other)	No
**vitamine**	2	Not classified (supplement/other)	No
**5 mg**	1	Not classified (supplement/other)	No
**75 resvega**	1	Not classified (supplement/other)	No
**acid folic**	1	Not classified (supplement/other)	No

**actonel saptamanal**	1	Osteoporosis therapy	No
**agnus**	1	Not classified (supplement/other)	No
**amlodipina**	1	Calcium-channel blockers	No
**anxiar**	1	SSRIs / benzodiazepine-related agents	Yes
**aplhad3**	1	Not classified (supplement/other)	No
**aprovel**	1	RAS agents (ACEi/ARB, incl. combinations)	No
**asacardin**	1	Antiplatelet agents	No
**aspenter 70 mg**	1	Antiplatelet agents	No
**aspenter alfa calcidol**	1	Antiplatelet agents	No
**aspenter75**	1	Antiplatelet agents	No
**aspirin cardio**	1	Antiplatelet agents	No
**atacand de 16**	1	RAS agents (ACEi/ARB, incl. combinations)	No
**atacand vessel due**	1	RAS agents (ACEi/ARB, incl. combinations); Venoactive agents	No
**atakand**	1	RAS agents (ACEi/ARB, incl. combinations)	No
**atoris**	1	Statins / lipid-lowering agents	No
**auronal**	1	Calcium-channel blockers	No
**b12**	1	Not classified (supplement/other)	No
**betaloc zok de 50 mg**	1	Beta-blockers	Yes
**betalok**	1	Beta-blockers	Yes
**betaser**	1	Other agents	No
**betaserg**	1	Other agents	No
**bilastina**	1	Antihistamines	Yes
**bisotens**	1	Beta-blockers	Yes
**bonium**	1	Not classified (supplement/other)	No
**calcidin**	1	Not classified (supplement/other)	No
**canzeno**	1	RAS agents (ACEi/ARB, incl. combinations)	No

**canzeno hct**	1	RAS agents (ACEi/ARB, incl. combinations); Diuretics (incl. fixed-dose combinations)	Yes
**capsule de ulei de salvie**	1	Not classified (supplement/other)	No
**caramlo 8**	1	RAS agents (ACEi/ARB, incl. combinations)	No
**carvedilol**	1	Beta-blockers	Yes
**cholestone**	1	Not classified (supplement/other)	No
**co prenesa**	1	RAS agents (ACEi/ARB, incl. combinations)	No
**co prenessa**	1	RAS agents (ACEi/ARB, incl. combinations)	No
**colagen**	1	Not classified (supplement/other)	No
**colagen magnézium**	1	Not classified (supplement/other)	No
**colesterol**	1	Not classified (supplement/other)	No
**concor 5mg**	1	Beta-blockers	Yes
**concor cor de 2.5**	1	Beta-blockers	Yes
**concor de 5**	1	Beta-blockers	Yes
**concor propafenonă**	1	Beta-blockers	Yes
**corlentor**	1	Other agents	No
**diaprel**	1	Antidiabetic agents	No
**diurex**	1	Diuretics (incl. fixed-dose combinations)	Yes
**duphaston**	1	Other agents	No
**eliquis**	1	Not classified (supplement/other)	No
**emgality**	1	Other agents	No
**escitalopram**	1	SSRIs / benzodiazepine-related agents	Yes
**etc**	1	Not classified (supplement/other)	No
**euthyrox 125 mg**	1	Levothyroxine	No
**euthyrox 50**	1	Levothyroxine	No
**eutirox 25 mg**	1	Levothyroxine	No
**everio**	1	Antihistamines	Yes

**exemestan**	1	Not classified (supplement/other)	No
**fac cure de colagen. nu am alte probleme de sănătate**	1	Not classified (supplement/other)	No
**femovita**	1	Not classified (supplement/other)	No
**fier**	1	Not classified (supplement/other)	No
**gerodorm**	1	SSRIs / benzodiazepine-related agents	Yes
**gingo biloba**	1	Not classified (supplement/other)	No
**gingobiloba**	1	Not classified (supplement/other)	No
**glucofage**	1	Antidiabetic agents	No
**hepatoprotector**	1	Not classified (supplement/other)	No
**hipertensiune**	1	Not classified (supplement/other)	No
**iau suplimente pentru simptome perimenopauza - vitex**	1	Not classified (supplement/other)	No
**indapamid**	1	Diuretics (incl. fixed-dose combinations)	Yes
**insulina**	1	Antidiabetic agents	No
**isicom**	1	Not classified (supplement/other)	No
**isoptin**	1	Calcium-channel blockers	No
**jardiance**	1	Antidiabetic agents	No
**levetipiracetam**	1	Antiepileptics	Yes
**levetiracetam**	1	Antiepileptics	Yes
**lipocomb**	1	Statins / lipid-lowering agents	No
**liver aid**	1	Not classified (supplement/other)	No
**mai iau ceva suplimente pentru memorie**	1	Not classified (supplement/other)	No
**medicamente pentru tensiunea**	1	Not classified (supplement/other)	No
**menopause**	1	Not classified (supplement/other)	No
**metformin**	1	Antidiabetic agents	No

**movymia**	1	Not classified (supplement/other)	No
**nebilet 5 mg**	1	Beta-blockers	Yes
**nebilet tertesi**	1	Beta-blockers; Diuretics (incl. fixed-dose combinations)	Yes
**noliprel de 2.5 mg**	1	RAS agents (ACEi/ARB, incl. combinations)	No
**noliprwl**	1	RAS agents (ACEi/ARB, incl. combinations)	No
**noliterax**	1	RAS agents (ACEi/ARB, incl. combinations)	No
**noriprel forte**	1	RAS agents (ACEi/ARB, incl. combinations)	No
**nu e cazul**	1	Not classified (supplement/other)	No
**nu e cazula**	1	Not classified (supplement/other)	No
**nu e xazul**	1	Not classified (supplement/other)	No
**ocazional - algocalmin**	1	Not classified (supplement/other)	No
**omega 3**	1	Not classified (supplement/other)	No
**oprymea**	1	Not classified (supplement/other)	No
**panangin**	1	Not classified (supplement/other)	No
**pentoxiretard**	1	Other agents	No
**pentru tensiune**	1	Not classified (supplement/other)	No
**physiotens**	1	Centrally acting antihypertensives	Yes
**plaquenil**	1	Not classified (supplement/other)	No
**plavix**	1	Antiplatelet agents	No
**preductal**	1	Not classified (supplement/other)	No
**prolia pt osteoporoza**	1	Osteoporosis therapy	No
**prolia ptr.osteoporoză**	1	Osteoporosis therapy	No
**propranolol**	1	Beta-blockers	Yes
**rhytmonorm**	1	Other agents	No

**roscord**	1	Statins / lipid-lowering agents	No
**rosuvastatina**	1	Statins / lipid-lowering agents	No
**rosvera**	1	Statins / lipid-lowering agents	No
**rovatinex**	1	Other agents	No
**rybelsus**	1	Antidiabetic agents	No
**serlift**	1	SSRIs / benzodiazepine-related agents	Yes
**sertlift**	1	SSRIs / benzodiazepine-related agents	Yes
**sevicar 20**	1	Not classified (supplement/other)	No
**sevikar**	1	RAS agents (ACEi/ARB, incl. combinations)	No
**simvacard**	1	Not classified (supplement/other)	No
**sortis 10 mg**	1	Statins / lipid-lowering agents	No
**spirulina**	1	Not classified (supplement/other)	No
**supliment hormonal ptr.hipotiroidism**	1	Not classified (supplement/other)	No
**suplimente alimentare**	1	Not classified (supplement/other)	No
**suplimente cu vitamine-orthomol**	1	Not classified (supplement/other)	No
**tanakan**	1	Not classified (supplement/other)	No
**vimovo**	1	Other agents	No
**vit d3**	1	Not classified (supplement/other)	No
**vit s**	1	Not classified (supplement/other)	No
**vitamina d 2000 u**	1	Not classified (supplement/other)	No
**vít d**	1	Not classified (supplement/other)	No
**xizal**	1	Antihistamines	Yes
**zi. ocazional magnerot**	1	Not classified (supplement/other)	No
**zomen**	1	RAS agents (ACEi/ARB, incl.	No

		combinations)
